# Visual impairment due to age‐related macular degeneration during 40 years in Finland and the impact of novel therapies

**DOI:** 10.1111/aos.15224

**Published:** 2022-08-01

**Authors:** Petri Purola, Kai Kaarniranta, Matti Ojamo, Mika Gissler, Hannu Uusitalo

**Affiliations:** ^1^ SILK, Department of Ophthalmology, Faculty of Medicine and Health Technology Tampere University Tampere Finland; ^2^ Finnish Register of Visual Impairment Finnish Federation of the Visually Impaired Helsinki Finland; ^3^ Department of Ophthalmology University of Eastern Finland and Kuopio University Hospital Kuopio Finland; ^4^ Department of Knowledge Brokers Finnish Institute for Health and Welfare Helsinki Finland; ^5^ Region Stockholm Academic Primary Health Care Centre Stockholm Sweden; ^6^ Department of Molecular Medicine and Surgery Karolinska Institute Stockholm Sweden; ^7^ Tays Eye Centre Tampere University Hospital Tampere Finland

**Keywords:** blindness, intravitreal drugs, vision loss

## Abstract

**Purpose:**

To evaluate the changes in visual impairment (VI) due to age‐related macular degeneration (AMD) during the past 40 years and the impact of novel therapies at population level.

**Methods:**

In this nationwide register‐based study, we assessed the incidence, prevalence, severity, and onset age of VI due to AMD based on the Finnish Register of Visual Impairment data from 1980 to 2019. Our data included 30 016 visually impaired persons with AMD as the main diagnosis for VI. The number of persons treated with intravitreal injections in Finland was obtained from hospital data kept by the Finnish Institute for Health and Welfare.

**Results:**

Between the 1980s and the 2010s, the incidence of reported VI doubled; however, this increase has stagnated in the 2010s. Since 2012, the prevalence of reported VI has decreased. The number of patients treated with intravitreal injections showed a 40‐fold increase during 2005–2019. The severity of reported VI has decreased whereas the mean age at the onset of reported VI has increased during the 40 years. The age‐adjusted incidence and prevalence of reported VI were significantly higher in females in comparison to males in all decades.

**Conclusion:**

Increase in the incidence and prevalence of VI due to AMD in the past decades has stagnated and shifted to older age in the 2010s when therapies for exudative class became commonly available. Furthermore, the prognosis of VI has improved during the past 40 years. These positive trends are likely contributable to improved diagnostic tools, earlier diagnoses, and new therapy options.

## 1 INTRODUCTION

Age‐related macular degeneration (AMD), which is mainly divided into exudative (wet) and non‐exudative (dry) classes, is the most common cause of visual impairment (VI) and blindness in developed countries, particularly in population aged 60 years or older (Bressler, 2004; GBD 2019 Blindness and Vision Impairment Collaborators & Vision Loss Expert Group of the Global Burden of Disease Study, 2021; Klaver et al., 1998; Klein et al., 1999; Mitchell et al., 2018; Wong et al., 2014). In US population aged 40 years or older, the estimated prevalence of any AMD was reported 6.5% and late AMD 0.8% (Klein et al., 2011). In Europe, the overall prevalence of early AMD was 13.2% and late AMD 3.0% in population aged 60 years or older (Colijn et al., 2017). In the Rotterdam Study, the prevalence of atrophic or neovascular AMD was 1.7%, and neovascular AMD was twice as common as atrophic AMD (Vingerling et al., 1995). In addition, the overall 2‐year cumulative incidence of AMD was 0.2%, increasing to 1.8% in persons aged 85 years or older (Klaver et al., 2001). In the Blue Mountains Eye Study, the prevalence of end‐stage AMD was 1.9%, rising from 0% among persons aged 55 years or younger to 18.5% among those aged 85 years or older (Mitchell et al., 1995). In Asia, the pooled prevalence estimates for populations aged 40–79 years were reported to be 6.8% for early AMD and 0.6% for late AMD (Kawasaki et al., 2010). The global prevalence of AMD among individuals 45–85 years is 8.7% (Wong et al., 2014). The projected number of people suffering from AMD in 2020 was 196 million, and it is estimated to increase to 288 million by 2040 (Wong et al., 2014). This increase is likely attributable to the ageing of populations (Bourne et al., 2014; Lindekleiv & Erke, 2013).

New therapy options for exudative AMD were developed in the 2000s. Photodynamic therapy (PDT) using verteporfin (V‐PDT; Visudyne, Novartis Ophthalmics) was approved in 2000 for the treatment of classic subfoveal choroidal neovascularization (Brown et al., 2006). Pegaptanib is an antivascular endothelial growth factor (VEGF) aptamer that binds to the isoform of VEGF‐165 (Macugen, Pfizer). It was approved in 2005 for the treatment of all exudative AMD subtypes (Gragoudas et al., 2004). Neither treatment resulted in clinically significant improvements in visual acuity (Brown et al., 2006; Gragoudas et al., 2004). Ranibizumab (Lucentis, Genentech and Novartis Ophthalmics), which was approved in 2006, is a humanized immunoglobulin GI (IgGI) antibody fragment against VEGF. Recombinant humanized monoclonal IgG1 antibody bevacizumab (Avastin, Genentech and Roche) was approved in 2004 for metastatic colorectal cancer but is used worldwide as an off‐label drug for exudative AMD (Zondor & Medina, 2004). VEGF‐trap aflibercept (Eylea, Bayer), a recombinant protein created by fusing the second Ig domain of human VEGF receptor 1 with the third domain of human VEGF receptor 2, which in turn is fused to the constant region of human IgG1, was approved 2011. Today, we can say that exudative AMD treatment has been revolutionized by the introduction of intravitreal anti‐VEGF drugs. Despite this development, undertreatment is frequent, and the availability of treatment and protocols differs between clinics (Holz et al., 2016; Klein & Klein, 2009; Martin et al., 2011; Monés et al., 2020; Schroeder et al., 2017; Tuuminen et al., 2017). Furthermore, non‐exudative AMD is still without effective treatment options. Therefore, it is important to evaluate the amount and burden of VI caused by the disease in order to improve health care and treatment.

Only few studies have evaluated the prevalence of AMD and its relation to VI and new therapy options (Bloch et al., 2012; Borooah et al., 2015; Bourne et al., 2014; Claessen et al., 2012; Colijn et al., 2017; Granstam et al., 2016; Skaat et al., 2012). Furthermore, these studies have usually been based on single‐centre recruitments and/or clinical trials with short follow‐up periods, and in many cases, the study population has only included persons with legal blindness due to AMD. Hence, there is a need for a comprehensive, population‐based evaluation of trends in VI due to AMD and the impact of new treatment options on the VI of AMD patients (Bourne et al., 2014). To fill this gap in knowledge, we investigated the changes in the incidence, prevalence, severity, and onset age of VI due to AMD during the past 40 years using nationwide, register‐based multicenter data collected from the Finnish Register of Visual Impairment during 1980–2019. We also investigated the potential impact of new therapies based on the number of persons treated with intravitreal injections collected from hospital data kept by the Finnish Institute for Health and Welfare during 1996–2019.

## 2 MATERIALS AND METHODS

### 2.1 Finnish Register of Visual Impairment and classification of visual impairment

The Finnish Register of Visual Impairment (The Finnish Federation of the Visually Impaired) was established in 1983, and its operation is regulated by the Act (556/89) and Decree (774/89) on National Personal Records kept under the Health Care System. Healthcare providers, specialists in ophthalmology, and the ophthalmological units of hospitals are, under the above‐mentioned Act, responsible to submit information on persons with permanent VI to the register without requiring permission from the patients. At the end of 2019, the register included data on 58 822 visually impaired persons. Registered data include eye diagnoses, date of birth, year of onset VI, and the classification of VI. The VI is classified according to the examination of ophthalmologists and the Finnish definitions of VI using visual acuity (VA) and visual field (VF) from central fixation (Ojamo, 2021), which are based on the definitions of World Health Organization (1973) with a modification of the nomenclature of the names of the VI classes here presented as Snellen decimals: (1) mild VI (0.3 > VA ≥ 0.1), (2) moderate VI (0.1 > VA ≥ 0.05), (3) severe VI (0.05 > VA ≥ 0.02; 10 > VF ≥ 5°), (4) near total blindness (0.02 > VA – 1/∞; VF <5°), and (5) total blindness (VA = 0; no sense of light). The time of VI is determined based on the notification data, and if it does not exist, the date of registration is used instead. The VI class is updated if any further information is notified.

### 2.2 Study population

In this study, we included visually impaired persons who had AMD as the main diagnosis of VI between 1980 and 2019. In addition, we received the number of patients treated with intravitreal injections (based on the NOMESCO Classification for Surgical Procedures code CKD05) in Finland during 1996–2019 from hospital data kept by the Finnish Institute for Health and Welfare. The annual population of Finland was provided by Statistics Finland. Age at death of the persons in the register was acquired from the Digital and Population Data Services Agency. This study was conducted in line with the tenets of the Helsinki Declaration. Because this is a register‐based study, the approval of ethical committee is not needed according to the Finnish legislation.

### 2.3 Statistical analyses

Annual incidence rates were calculated by dividing the number of new registered persons with the number of inhabitants per year by sex and age group. Annual prevalence rates were calculated similarly using cumulatively summed registered persons alive at the end of the year. For incidence rates, we also calculated average annual rates per decade adjusted for age and sex. We used linear regression to compare annual‐based trends and chi‐squared test to compare decade‐based trends. The expected number of years with VI was calculated by subtracting the mean age at the onset of reported VI from the mean age at death in each decade. Because age data was left‐skewed, Mann–Whitney *U* test was used for between‐group comparisons. A two‐tailed *p* value of <0.05 was considered statistically significant. All statistical analyses were performed using r software version 4.1.1 (R Core Team, Foundation for Statistical Computing, Vienna, Austria).

## 3 RESULTS

The Finnish Register of Visual Impairment included a total of 30 016 (70.5% females) visually impaired persons with AMD registered as the main diagnosis of VI at the end of 2019. Of these persons, 3913, 6806, 9332, and 9965 had become visually impaired in the 1980s, 1990s, 2000s, and 2010s, respectively. The share of females in each decade was 71.7%, 70.3%, 71.2%, and 69.4%, respectively. At the end of 2019, the population of Finland was 5 525 292 (50.6% females), of which 1 231 274 (56.0% females) were aged 65 years or older.

The most common class of AMD to cause VI was non‐exudative AMD (43.8%), followed by exudative AMD (28.2%) and unspecified AMD (28.0%). Among females the distribution of AMD classes was 43.3%, 28.8%, and 27.9%, respectively, and among males 45.0%, 29.1%, and 25.9%. Unspecified AMD was significantly more common among females (*p* < 0.001); however, the number of unspecified diagnoses had decreased in the 2010s due to the improved classification of AMD.

**FIGURE 1:**
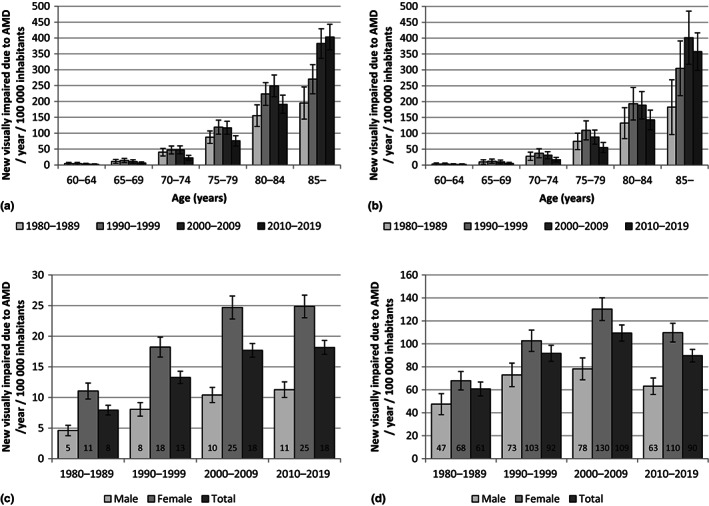
Average annual incidence of reported visual impairment due to age‐related macular degeneration (AMD) per 100 000 female (a) and male (b) inhabitants per decade, and average annual incidence in total population (c) and population aged 65 years or older (d) per decade with 95% confidence intervals. In (c), male and female incidences were adjusted for age, and total incidences were adjusted for age and sex.

**FIGURE 2:**
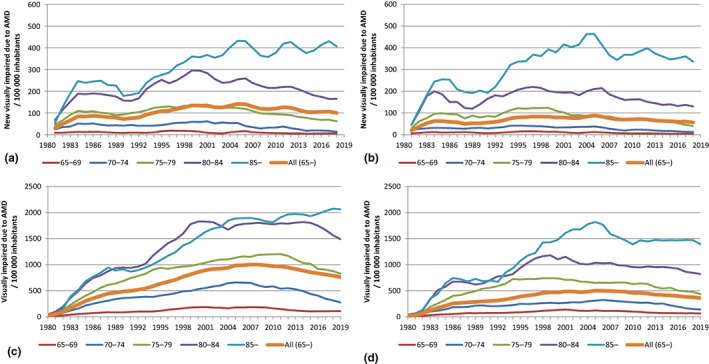
Annual incidence (a, b) and prevalence (c, d) of reported visual impairment due to age‐related macular degeneration (AMD) per 100 000 female (a, c) and male (b, d) inhabitants. Incidences (a, b) were smoothed using a three‐year central moving average.

The incidence of reported VI due to AMD in the Finnish population in the four decades by age and sex is shown in Figure 1a,b. Regarding total population (Figure 1c), the annual age‐ and sex‐adjusted incidence of reported VI due to AMD per 100 000 inhabitants had significantly increased from 7.9 (95% confidence interval [CI] 7.1–8.7) in the 1980s to 18.2 (95% CI 17.0–19.3) in the 2010s (*p* < 0.001). This increase was significant in both sexes (*p* < 0.001). During the 40 years, the incidence had increased most noticeably in the age group 85 years or older in both sexes (Figures 1a,b, 2a,b). There was no significant difference in the incidence between the 2000s and the 2010s in either sex (Figure 1c). Regarding population aged 65 years or older (Figures 1d, 2a,b), the incidence of reported VI due to AMD had significantly increased from the 1980s to the 2010s in both sexes (*p* < 0.001), but the incidence had significantly decreased between the 2000s and the 2010s in both females (*p* = 0.002) and males (*p* = 0.014) aged 65 years or older. There was no significant difference in the incidence between the 1990s and 2000s in males aged 65 years or older (Figure 1d). The incidence was significantly higher in females in each decade in total population and population aged 65 years and older (*p* < 0.001).

When the prevalence of reported VI due to AMD was observed in population aged 65 or older, there was a significant increase in the prevalence during 1980–2012, but it has since significantly decreased (Figure 2c,d; *p* < 0.001). Only in females aged 85 years or older, the prevalence has significantly kept increasing in the 2010s (*p* < 0.001), and in males aged 85 years or older the prevalence has not significantly changed since 2010. The annual prevalence was significantly higher in females in total population and population aged 65 years or older (*p* < 0.001).

**FIGURE 3:**
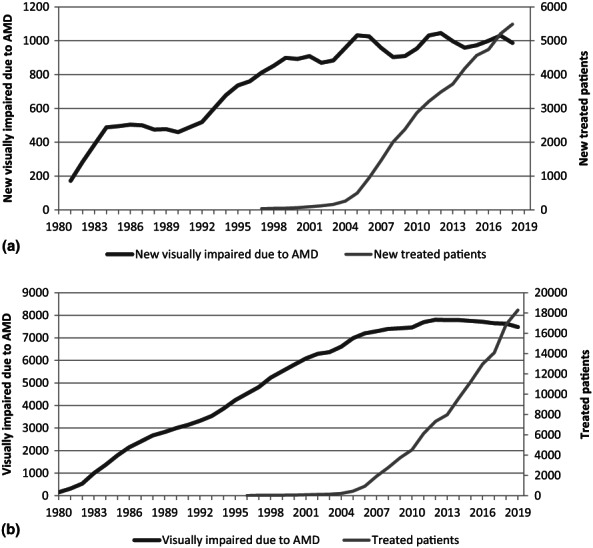
Number of new (a) and all (b) persons with reported visual impairment due to age‐related macular degeneration (AMD) and persons treated with intravitreal injections per year. Incidences (a) were smoothed using a three‐year central moving average.

The number of persons with reported VI due to AMD and patients treated with intravitreal injections per year is shown in Figure 3. The number of all treated patients per year increased from 448 in 2005 to 18 286 in 2019, and the respective numbers for new cases per year were 418 and 5545. During the same period, the increase in VI had stagnated and the prevalence started to decrease in total population.

The distribution of reported VI classes is shown in Table 1. The percentage of mild VI had significantly increased from 63% in the 1980s to 78% in the 2010s (*p* < 0.001). The shares of other more severe classes of VI had decreased. No statistically significant differences between sexes were found in any of the decades.

**TABLE 1:** Distribution of reported visual impairment due to age‐related macular degeneration

Visual impairment	1980–1989	1990–1999	2000–2009	2010–2019
Mild visual impairment	62.9%	68.5%	73.2%	78.0%
Moderate visual impairment	19.5%	18.2%	16.5%	12.6%
Severe visual impairment	14.8%	9.4%	7.7%	7.5%
Near total blindness	2.7%	3.9%	2.6%	1.9%
Total blindness	0.1%	0.0%	0.0%	0.0%

The age at the onset of reported VI by decade of onset is shown in Table S1A. The mean age had significantly increased from 78.3 years (95% CI 78.1–78.6) in the 1980s to 84.0 years (95% CI 83.9–84.2) in the 2010s (*p* < 0.001). The mean age among females had significantly increased from 78.8 years (95% CI 78.6–79.1) to 84.5 years (95% CI 84.4–84.7) during the same period. Among males the mean age had significantly increased from 77.1 years (95% CI 76.6–77.6) to 82.9 years (95% CI 82.6–83.2) during the same period (p < 0.001). Females had significantly higher mean age compared to males in each decade (*p* < 0.001).

The age at death in persons with reported VI due to AMD by decade of onset is shown in Table S1B. The mean age had significantly increased from 86.8 years (95% CI 86.6–87.0) in the 1980s to 89.5 years (95% CI 89.2–89.7) in the 2010s (*p* < 0.001). The mean age among females had significantly increased from 87.6 years (95% CI 87.3–87.8) to 90.3 years (95% CI 90.0–90.5) during the same period (*p* < 0.001), and among males the numbers were 84.9 (95% CI 84.5–85.3) and 87.9 (95% CI 87.6–88.3) (*p* < 0.001). Females had significantly higher mean age compared to males in each decade (*p* < 0.001).

**FIGURE 4:**
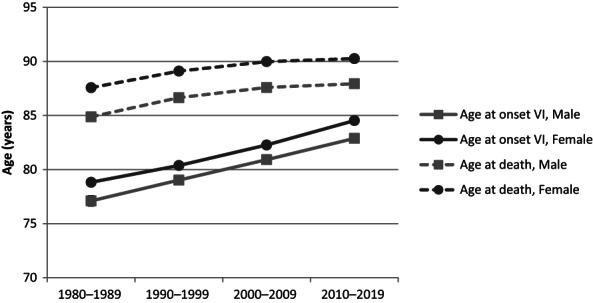
Age at the onset of reported visual impairment (VI) due to age‐related macular degeneration and age at death by decade of onset. 95% confidence intervals are included but are not visible due to their small size.

The age at the onset of reported VI and the age at death are illustrated in Figure 4. The expected number of years with VI had significantly decreased in females from 8.8 years in the 1980s to 5.7 years in the 2010s (*p* < 0.001). In males, respective numbers were 7.8 and 5.1 (*p* < 0.001).

## 4 DISCUSSION

In this study based on population‐level register data, we show that the incidence and prevalence of VI due to AMD have increased between the 1980s and the 2010s; however, the increase in incidence has stagnated in the 2010s and the prevalence started to decrease in the same decade. During the same period these changes have occurred, the number of persons treated with intravitreal injections has dramatically increased. These positive trends were noticeable in all age groups except those aged 85 years and older, indicating a shift to older age. This shift is also indicated by the delaying of the age at the onset of VI due to AMD. Our results also show that the severity of VI has decreased during the 40 years.

There are two likely explanations for the increase in the incidence and prevalence of VI due to AMD between the 1980s and the 2010s. First, the ageing of the population is likely to increase the prevalence and incidence of AMD and the subsequent VI (Colijn et al., 2017; Klein & Klein, 2009). People living past 80 years are particularly making up a larger proportion of the population, as shown in our data. Second, many improvements, such as developed imaging technology and understanding of pathogenesis, have occurred in the detection and diagnosis of AMD during the past decades, likely contributing to the increasing number of registered AMD patients.

What was most remarkable, however, was the stagnation of the incidence and prevalence in the 2010s – in fact, even some decline was observed in younger age groups. Granstam et al. (2016) reported a similar decline in the incidence of severe VI attributable to AMD between 2005 and 2013; however, the study included only three time points and persons from low vision clinics in five counties in northern Sweden. Colijn et al. (2017) also observed a decrease in VI due to AMD after 2006 in a meta‐analysis based on 14 population‐based cohorts from 10 countries in Europe between 1990 and 2013.

Bloch et al. (2012) reported a reduction of 50% in legal blindness due to AMD between 2000 and 2010 in Denmark, the bulk of the reduction occurring after 2006. Skaat et al. (2012) reported significant decline in the incidence of blindness due to AMD between 1999 and 2008 in Israel. The greatest decline in the incidence was observed in persons aged 65 years or younger. Claessen et al. (2012) observed decline in the incidence of blindness due to AMD in 2008–2009 in Germany when compared to previous study in 1994–1998. These results support our findings; however, all three studies included only a 10‐year follow‐up and persons with (legal) blindness. A meta‐analysis based on population‐based surveys showed that the estimated global prevalence of blindness due to AMD declined by almost 30% from 1990 to 2020; however, the authors pointed out that these figures are estimates of regional‐level data, which can be biased due to the diversity of existing situations within countries and communities (GBD 2019 Blindness and Vision Impairment Collaborators & Vision Loss Expert Group of the global burden of disease study, 2021).

These studies have suggested that the decline in the VI due to AMD is because of healthier lifestyles and the implementation of anti‐VEGF treatment since the mid‐2000s. In addition, many public campaigns have likely made elderly persons more aware of the symptoms and new therapy options, and therefore have contributed to the decrease in VI (Bertram et al., 2014; Heraghty & Cummins, 2012). Interestingly, in a study based on a systematic review of medical literature, Bourne and colleagues reported a significant increase in the proportion of blindness due to AMD between 1990 and 2010 in high‐income countries (Bourne et al., 2014). They suggested that this negative change is possibly due to an increased life expectancy leading to an increased prevalence of AMD and that it may reflect the challenges in treatment of AMD (Bourne et al., 2014; Colijn et al., 2017; Wong et al., 2014).

In Finland, anti‐VEGF treatments are based on Finnish national guidelines for exudative AMD that allow fixed, pro re nata, and treat‐and‐extend protocols and bevacizumab, ranibizumab, and aflibercept intravitreal injections (Tuuminen et al., 2017). The guideline recommends starting anti‐VEGF treatment with bevacizumab and continue with it as long as treatment response can be observed. The indication to switched treatment to a second drug is usually done once bevacizumab is not effective enough. The decision to switched drug could be because visual acuity did not improve, the optical coherent tomography failed to show an effect on retinal fluid, or the interval between injections could not be extended beyond four weeks. Most switches are made to aflibercept in line with previous studies (Barthelmes et al., 2016, 2018; Karesvuo et al., 2020; Verbraak et al., 2020). The use of ranibizumab in Finland has been very marginal all the time. Here we show decreased VI due to AMD after 2010. The delay in improved VI prevention may be due to lack awareness of the therapeutic options among population and primary health care as well as lack of experience to control and treat exudative AMD during the first years when anti‐VEGF intravitreal drugs were approved. PDT was used routinely in Finnish ophthalmology centers during 2000–2005, but as known, its capacity to improve visual acuity remained weak (Brown et al., 2006). Pegabtanib was used in Finland for a short period, until more effective bevacizumab and ranibizumab replaced it immediately when they became possible to use. According to Finnish guidelines of AMD, bevacizumab is the first‐line therapy, but it is switched to aflibercept once required to prevent VI. It is also noteworthy that during the couple past years many centers have started with aflibercept as first‐line therapy due to less clinic visit requirements. However, one can estimate that this does not affect the current data.

Our study shows that the incidence and prevalence of VI due to AMD are more common among females than males. Similar trend has been shown in global scale, as the estimated age‐standardized global prevalence of blindness due to AMD was found greater in females (GBD 2019 Blindness and Vision Impairment Collaborators & Vision Loss Expert Group of the global burden of disease study, 2021). Gender differences have been reported in the health behaviour in general (Mahalik et al., 2006; Weber et al., 2019) and in Finland particularly (Koponen et al., 2018; Vaajanen et al., 2021). Therefore, the undertreatment of exudative AMD among females is not explaining this difference. Females have longer life expectancy than males in Finland and therefore are more susceptible to diseases associated with age, such as AMD (Koponen et al., 2018). The longer life expectancy of females is likely to also explain the higher age at the onset of VI in comparison to males. Nevertheless, as females showed significantly higher age‐adjusted incidence and age‐stratified prevalence than males, and as there were no significant differences in VI grades between sexes, this suggests that VI due to AMD is indeed more common among females.

AMD has been associated with severe effects on quality of life, including greater life stress, lower activity in daily living, and increased depression (Brody et al., 2001; Gopinath et al., 2014; Mitchell & Bradley, 2006; Purola et al., 2021). This is likely due to the VI associated with the disease (Purola et al., 2021). Even though the recent treatment possibilities for the exudative AMD have had a reducing effect on the VI and the time the AMD patients are living visually impaired has decreased as shown in this study, frequent hospital visits due to the treatment and the disease itself likely keep having impact on the patient's quality of life. Furthermore, non‐exudative AMD remains a significant cause for VI in Finland and other developed countries. Hence, there is a need to improve existing treatment options for exudative AMD and develop new therapies for non‐exudative AMD to reduce negative impact on quality of life of the patients, burden of health care, and the direct and indirect costs to society.

Our study had many strengths. We had a unique possibility to use nationwide registers that represent population well, as similar registers are rare worldwide. We had high number of data based on routinely collected health registers, and therefore our results are comparable with those from studies in the other developed countries. We also had access to data from four decades, giving us a relatively large timescale of 40 years. The classification of VI is based on the Finnish national definitions and recommendations modified from the World Health Organization (1973) definitions, which cover both decreased visual acuity and visual field constriction.

Our study also had limitations. Visual impairment register data, like register data in general, can have potential sources of biases. It is difficult to estimate the exact time point at which a person becomes visually impaired, and it is even more difficult to estimate when the disease itself emerges. Because aged patients may suffer from more than one vision‐threatening disease, we analysed only those patients whose main diagnosis causing VI was AMD. We could not analyse exudative and non‐exudative data separately due to the relatively high number of unspecified AMD diagnoses in the 1980s, 1990s, and 2000s. The register may lack information on specific populations, such as institutionalized demented persons. Our data included predominantly people with Finnish background, and therefore our results may not be directly applicable to other countries and ethnicities.

In conclusion, our population‐based register data show that the incidence and prevalence of VI due to AMD have increased during the past 40 years due to the ageing of population. This trend appears to have stagnated or decreased in the 2010s and shifted to older age. The severity of VI and expected number of years with VI have also decreased during the past 40 years. These positive trends are likely due to the modern treatment possibilities for exudative AMD. Despite these recent developments, non‐exudative AMD remains a significant cause for VI in developed countries.

## Supporting information


**Table S1** (A) Age at the onset of reported visual impairment due to age‐related macular degeneration by decade of onset. (B) Age at death in persons with reported visual impairment due to age‐related macular degeneration by decade of onset
