# Supplementary material for: Visual impairment due to age‐related macular degeneration during 40 years in Finland and the impact of novel therapies
Source: Acta Ophthalmol. 2022 Aug 1;101(1):57–64. doi: 10.1111/aos.15224 (PMC10087211; doi:10.1111/aos.15224)
Supplement: Supplementary file 1 — Table S1 (A) Age at the onset of reported visual impairment due to age‐related macular degeneration by decade of onset. (B) Age at death in persons with reported visual impairment due to age‐related macular degeneration by decade of onset [file AOS-101-57-s001.docx]

Table S1A. Age at the onset of reported visual impairment due to age-related macular degeneration by decade of onset

|  | 1980–1989 | 1990–1999 | 2000–2009 | 2010–2019 |
| --- | --- | --- | --- | --- |
| Male |  |  |  |  |
| n | 1108 | 2021 | 2688 | 3048 |
| Mean, years  (95% CI) | 77.1  (76.6–77.6) | 79.0  (78.7–79.4) | 80.9  (80.6–81.2) | 82.9  (82.6–83.2) |
| Female |  |  |  |  |
| n | 2805 | 4785 | 6644 | 6917 |
| Mean, years (95% CI) | 78.8  (78.6–79.1) | 80.4  (80.2–80.6) | 82.3  (82.1–82.4) | 84.5  (84.4–84.7) |

There was a statistically significant difference in age between sexes in each decade (p < 0.001, Mann–Whitney U test). CI, confidence interval.

Table S1B. Age at death in persons with reported visual impairment due to age-related macular degeneration by decade of onset

|  | 1980–1989 | 1990–1999 | 2000–2009 | 2010–2019 |
| --- | --- | --- | --- | --- |
| Male |  |  |  |  |
| n | 1100 | 1971 | 2392 | 1398 |
| Mean, years (95% CI) | 84.9  (84.5–85.3) | 86.6  (86.3–86.9) | 87.6  (87.3–87.9) | 87.9  (87.6–88.3) |
| Female |  |  |  |  |
| n | 2791 | 4663 | 5718 | 2703 |
| Mean, years (95% CI) | 87.6  (87.3–87.8) | 89.1  (88.9–89.3) | 90.0  (89.8–90.1) | 90.3  (90.0–90.5) |

There was a statistically significant difference in age between sexes in each decade (p < 0.001, Mann–Whitney U test). CI, confidence interval.
